# Balancing standardisation and individualisation in transitional care pathways: a meta-ethnography of the perspectives of older patients, informal caregivers and healthcare professionals

**DOI:** 10.1186/s12913-022-07823-8

**Published:** 2022-04-01

**Authors:** Linda Aimée Hartford Kvæl, Ragnhild Hellesø, Astrid Bergland, Jonas Debesay

**Affiliations:** 1 Faculty of Health Sciences, Department of Physical Therapy, Oslo Metropolitan University, PO box 4 , St. Olavs plass, NO-0130 Oslo, Norway; 2 Norwegian Social Research - NOVA, Department of Ageing Research and Housing Studies, Oslo Metropolitan University, PO box 4, St. Olavs plass, NO-0130 Oslo, Norway; 3 Faculty of Medicine, Institute of Health and Society, Department of Nursing Science, University of Oslo, P.O. 1130, Blindern, 0318 Oslo, Norway; 4 Center for Care Research, Norwegian University of Science and Technology (NTNU), Pb 191, 2802 Gjøvik, Norway; 5 Faculty of Health Sciences, Department of Nursing and Health Promotion, Oslo Metropolitan University, PO box 4, St. Olavs plass, NO-0130 Oslo, Norway

**Keywords:** Meta-ethnography, Person-centred care, Transitional care, Older persons, Clinical work, Integrated care, Norway

## Abstract

**Background:**

Transitional care implies the transfer of patients within or across care settings in a seamless and safe way. For frail, older patients with complex health issues, high-quality transitions are especially important as these patients typically move more frequently within healthcare settings, requiring treatment from different providers. As transitions of care for frail people are considered risky, securing the quality and safety of these transitions is of great international interest. Nevertheless, despite efforts to improve quality in transitional care, research indicates that there is a lack of clear guidance to deal with practical challenges that may arise. The aim of this article is to synthesise older patients, informal caregivers and healthcare professionals’ experiences of challenges to achieving high-quality transitional care.

**Methods:**

We used the seven-step method for meta-ethnography originally developed by Noblit and Hare. In four different but connected qualitative projects, the authors investigated the challenges to transitional care for older people in the Norwegian healthcare system from the perspectives of older patients, informal caregivers and healthcare professionals. In this paper, we highlight and discuss the cruciality of these challenging issues by synthesising the results from twelve articles.

**Results:**

The analysis resulted in four themes: i) balancing person-centred versus efficient care, ii) balancing everyday patient life versus the treatment of illness, iii) balancing user choice versus “What Matters to You”, and iv) balancing relational versus practical care. These expressed challenges represent tensions at the system, organisation and individual levels based on partial competing assumptions on person-centred-care-inspired individualisation endeavours and standardisation requirements in transitional care.

**Conclusions:**

There is an urgent need for a clearer understanding of the tension between standardisation and individualisation in transitional care pathways for older patients to ensure better healthcare quality for patients and more realistic working environments for healthcare professionals. Incorporating a certain professional flexibility within the wider boundary of standardisation may give healthcare professionals room for negotiation to meet patients’ individual needs, while at the same time ensuring patient flow, equity and evidence-based practice.

## Background

Better and safer healthcare transitions are an international priority [1]. Transitional care encompasses the transfer of patients within or across care settings, that is, vertically or horizontally, in a seamless and safe way [2]. The safety of older persons might be threatened during care transitions from specialist to primary care, or within levels, and research supports that these transitions have the potential for improvement [3]. Increasingly specialised healthcare service, in combination with a trend of decentralisation of tasks from specialist care to the municipalities, means that persons are discharged from hospitals earlier than before [4]. For older patients with complex health issues, high-quality transitions are crucial to promote integrated care as these patients typically move more frequently within healthcare settings [5]. Even though there are differences in ways of understanding integrated healthcare, Goniewicz et al. (2021) describe it as services that make humans healthy [6], while according to the World Health Organisation (WHO), an integrated system implies a healthcare service organised and managed in order to deliver the right care at the right place in a timely manner and in a patient-friendly way, while promoting patient outcomes in a cost-efficient way [7].

Transitional care pathways might be complicated and multifaceted, consisting of various designs and functions, embraced by both promoting and inhibiting mechanisms [8]. Patient handover is a key aspect in transitional care and implies the handover of essential information, communication between involved healthcare professionals (HCPs) and the overall transfer of care responsibility to another care level [9]. In four different but connected empirical projects (https://uni.oslomet.no/crosscareold/), we explored the challenges of quality transitional care for older people in the Norwegian healthcare context from the views of older persons, informal caregivers and HCPs. This article synthesises the findings across all four sub-projects to produce a greater understanding.

### High-quality transitional care

Key determinants in high quality transitional care are the preparation of patients and informal caregivers for care transitions, active involvement of patients and informal caregivers in plans of care, communication between professionals, follow-up plans, systems that facilitate transitional care and education of HCPs [10]. Locations of care might be hospitals, subacute and post-acute units, patients’ homes, assisted living facilities and nursing homes. Overall, the success of transitional care relies on holistic care transitions incorporating both the hospital discharge process and follow-up services [11], which requires that the staff know the patient, know each other and thus bridge the gap in the system [12].

A strong research focus has been to identify risk factors related to hospital readmission: i) the older person’s demographics, such as higher age, male gender, living conditions and ethnicity, ii) health characteristics, such as morbidity, dysfunctionality and prior admissions and iii) the context of care, such as length of stay, method of referral and discharge location [13, 14]. Glans et al. (2020) show in their recent systematic review and meta-analysis that being discharged on a Friday or from a surgical unit increases the risk of readmission [15]. Other challenges are discontinuity between HCPs working in specialist and primary healthcare, changes to medications, self-management duties that may place stress on current resources and complicated discharge procedures [16]. Challenges to achieving collaboration between care levels due to individual and/or organisational structures may result in inadequate healthcare services and lengthen institutional stays for older persons [17]. According to the WHO and the European parliament, in order to be prepared to accommodate the growing frail population, healthcare systems have to change. Healthcare system transitions should aim to more firmly anchor primary care as a cornerstone of healthcare systems because of the long-term relationships between primary HCPs and older patients [18].

Transitional care programmes have been developed to improve the quality of transitional care with the objective of obtaining coordinated care in the transition from hospital to other locations. In Europe and the UK, these schemes are called intermediate care (IC) models. Intermediate care includes the services that facilitate the transitions from hospital to home and the recovery from medical and social dependence to functional independence [19]. You can be provided with IC services in municipal institutions, in nursing homes, in special hospital units, or in the patient’s home [20]. In Canada and Australia the focus is on transitional care programmes, while in the US transitional care programmes are also supplemented by sub- and post-acute care, e.g. skilled nursing facilities [21]. In common, transitional care programmes are designed to provide short-term care and facilitate transitions between care levels [19–22]. Nevertheless, despite efforts to improve quality in transitional care, research indicates that there is a lack of clear guidance to deal with the practical challenges that may arise [23].

### Person-centred integrated care

Due to the multidimensional nature of multimorbidity, it is of great relevance that the healthcare provided is holistic and that the clinical pathway is not only based on single diagnoses. People with multimorbidity, that is, two or more diagnoses [24], need person-centred care (PCC), a model in which HCPs need to develop a certain skill mix [25]. In Western countries, there is a growing acknowledgement that quality health services should be effective, safe and person-centred, as well as timely, equitable, integrated and efficient [26]. PCC is highlighted by the Institute of Medicine (2001) as respectful care that is responsive to a patient’s preferences and needs, ensuring that the patient’s values are the guiding principle in clinical decisions [27]. In their concept analysis, Morgan and Yoder (2012) describe the defining attributes of PCC in post-acute care as holistic, individualised and empowering [28].

The WHO emphasises both PCC and integrated care as crucial aspects of quality healthcare in persons with multimorbidity [29]. Research shows that many older people are ‘falling through the gaps’ and experiencing fragmented healthcare, particularly when living with frailty [30]. PCC implies putting older patients at the centre of their care by involving them in decisions, as well as seeing the person behind the diagnosis in a holistic way while treating them with respect and dignity [31]. In addition, the services provided to frail, older patients must be coordinated [29, 32, 33]. Integrated care includes ‘structured efforts to provide coordinated, pro-active, person-centred, multidisciplinary care by two or more well-communicating and collaborating care providers either within or across sectors’ ([33] p. 13). To achieve integrated care, a move towards a more PCC model is called for in service provision, leadership and even financial systems [34], meaning that person-centred integrated care must be delivered at all levels, including macro, meso and micro levels [31, 33, 35]. Therefore, high-quality transitional care should be recognised as an important aspect of integrated care [36].

### Aims of the study

Research within transitional healthcare has to a large extent consisted of evaluating readmission rates, prioritising quality domains such as efficiency for inpatient providers, risk and safety. We believe this is the first article to synthesise knowledge regarding users’ experiences about challenges in high-quality transitional healthcare. Therefore, there is still a lack of evidence within quality dimensions like punctuality, seamless care, fairness, efficiency for HCPs, success of self-management interventions and various aspects of PCC, suggesting that users’ experiences have not received sufficient attention [37]. Hence, the overall aim of this article is to synthesise older patients, informal caregivers and HCPs’ experiences of challenges to achieve high-quality transitional care using meta-ethnography. The method is appropriate to produce a greater insight than the individual parts can provide alone [38].

## Methods

We have used the seven-step method for conducting meta-ethnography originally developed by Noblit and Hare [38]. Meta-ethnography aims to create an interpretive integration of primary qualitative research reports. The four sub-projects representing 12 articles (Table 1) included in this meta-ethnography address the same phenomenon of challenges experienced to achieve high-quality transitional care, thus constituting a reciprocal translation approach.

**Table 1:** Overview of the 12 included articles in the meta-ethnography

Article	Aim	Main themes
1. Hestevik et al. (2019) [39]	‘To explore how HCPs experience providing individualized nutritional care within the organizational frames of acute geriatric hospital care and home care’ ([39] p. 2)	1) Meeting patients with complex nutritional problems 2) The structure of nutritional care
2. Hestevik et al. (2020) [40]	‘To explore older patients’ and family caregivers’ perceptions regarding the food, meals and nutritional care provided in the transition between hospital and home the first 30 days at home’ ([40] p. 2)	1) The need for a comprehensive approach to nutritional care 2) Non-individualised nutritional care at home 3) Lack of mutual comprehension and shared decision making 4) The role of family caregivers
3. Hestevik et al. (2020) [41]	‘To explore HCPs’ views on how older persons and their family caregivers participate in decisions about their own nutritional care and possible barriers for that participation’ ([41] p. 199)	1) Lack of shared decision making in nutritional care 2) Conflict between patients’ preferences and standard nutritional care procedures 3) The value of family caregivers who are seldom involved in nutritional care
4. Kvæl et al. (2019) [42]	‘To explore older patients’ and their relatives’ experiences and preferences regarding patient participation in IC and identify types of patient participation and their potential empowering or disempowering effect’ ([42] p. 2)	1) Lack of choice and expectation of compliance 2) The need of a rehabilitation perspective and reciprocal engagement 3) Patient participation meeting experts’ views
5. Kvæl et al. (2019) [43]	‘To explore how HCPs, experience patient participation in IC, and explain how they perform their clinical work balancing between the patients’ needs, resources and regulatory constraints’ ([43] p. 923)	1) The purchaser-provider model and standardisation of patient participation 2) Intermediate care as a storage facility losing its rehabilitative function 3) Lack of professional discretion and empowerment of HCPs
6. Kvæl et al. (2020) [44]	‘To explore the negotiation of patient participation in family meetings in IC services, by observing the interactions between the older patient, their relatives, and the HCPs in IC’ ([44] p. 812)	1) Patients’ needs for masquerade to participate 2) The strategies of relatives in coming across 3) Professionals’ roles in defining the situation
7. Lilleheie et al. (2020) [45]	‘To explore the experiences of older patients and informal caregivers in the first 30 days after the patient’s discharge with a special focus on the burden of care’ ([45] p. 2)	1) Bridging the gap 2) Family is family 3) Never enough 4) Stress and distress
8. Lilleheie et al. (2020) [46]	‘To explore older patients’ informal caregivers’ views on healthcare quality in the hospital and in the first 30 days after hospitalisation’ ([46] p. 2)	1) Fast in, fast out 2) Scant information 3) Disclaimer of responsibility 4) The struggle to secure professional care
9. Lilleheie et al. (2020) [47]	‘To explore older patients’ experiences of the quality of the health services in hospital and the first 30 days at home after discharge’ ([47] p. 2)	1) Hospital stays and the person behind the diagnosis 2) Poor communication and coordination 3) Life after discharge 4) Patients’ relationship with their next of kin 5) Organisational and systemic determinants
10. Olsen et al. (2020) [48]	‘To explore health care providers’ perceptions and experiences regarding “What matters to you?” in the context of improving transitional care for older, chronically ill persons’ ([48] p. 3)	1) WMTY [what matters to you] is a complex process that needs to be framed competently 2) Framing WMTY as a functional approach 3) Framing WMTY as a relational approach
11. Olsen et al. (2021) [49]	‘To explore HCPs’ experiences and understandings of implementing a care pathway to improve the quality of transitional care for older people’ ([49] p. 3)	1) Understanding the care pathway as patient flow 2) Understanding the care pathway as the patient’s journey 3) The dilemma between improving patient flow and the patient’s journey
12. Olsen et al. (2021) [50]	‘To explore HCPs’ perceptions and experiences of what is important to achieve more person-centered patient pathways for older people’ ([50] p. 3)	1) Finding common ground through the mapping of the patient journey 2) The importance of understanding the whole patient pathway 3) The significance of getting to know the older patient 4) The key role of home care providers in the patient pathway 5) Ambiguity towards checklists and practice implementation

The seven-step method includes the following stages: 1) getting started, 2) deciding what is relevant to the initial interest, 3) reading the studies, 4) determining how the studies are related, 5) translating the studies into one another, 6) synthesising the translations and 7) expressing the synthesis. The process is iterative, and the phases may overlap [38]. We have followed the step-by-step guideline by Sattar et al. [51] and the eMERGe meta-ethnography reporting guidance by France et al. [52].

### Setting

The Norwegian healthcare system is largely public and separated into the specialised and primary care healthcare levels. Specialised healthcare includes state-owned hospitals, arranged into four geographical health authorities, while primary care includes home-based services and nursing home facilities [53]. Due to demographic developments and specialised care services, increased attention to integrated care models has evolved [54]. Hence, Norway has, over the last two decades, developed IC services based on different variants of shared care between specialised and primary care [55] and introduced a daily penalty fee if primary care does not have a suitable service to older persons ready for hospital discharge [17]. In Norway, similar to other industrialised states, new public management ideas of how to organise the healthcare system obtained huge political influence beginning in the 1990s [56]. This priority on cost-effectiveness, as well as deinstitutionalisation, has led to high turnover rates of hospital patients and an increased workload in the municipalities [57] through an emphasis on measuring outcomes and quasi-market solutions [17]. For example, a purchaser-provider model has been developed [58, 59]. This implies a distinction between the municipal administration having the authority and the HCPs who work with patients face to face having little influence on the patient pathway [57]. For example, after hospitalisation, the municipal district coordinator approves home-based services where the home staff provide the work based on written, predefined time-limited goals. Furthermore, the policy of ‘ageing in place’ supports the opportunity for older persons to stay at home for as long as possible to prevent long-term care and meet the needs of older patients while supporting their quality of life [60]. However, political reforms and regulations emphasise older patients’ needs for improved and more coordinated healthcare services [54, 61]. Therefore, healthcare services should set the patient at the centre of care and deliver proper care in the right place at the right time [54, 62].

### Material

This meta-ethnography is based on the larger project ‘Cross-Care-Old: A Cross-Sectoral Approach to High-Quality Healthcare Transitions for Older Persons’, undertaken from 2016–2021 in Norway (https://uni.oslomet.no/crosscareold/). The purpose of Cross-Care-Old was to generate new insights on cross-sectoral healthcare transitions for older persons and relatives. One part of the Cross-Care-Old project incorporated the experiences and preferences of geriatric patients, informal caregivers and HCPs from four different but interlinked PhD projects: i) nutritional care in the transitions between specialist and primary care, ii) patient participation in IC services, iii) quality of hospital healthcare and 30 days after discharge and iv) experiences from a Norwegian quality improvement collaborative. The twelve articles constituting the empirical material in this synthesis are outlined in Table 1.

### Getting started—Phase 1

Phase 1 required that the authors identify an area of interest [38], which in our case was the challenges to achieving high-quality transitional care for geriatric patients. In the background section, we have accounted for the rationale and context for the meta-ethnography (research and knowledge gap), aims and focus of the synthesis, as well as the rationale for choosing meta-ethnography as the methodology [52]. Further, in order to ensure an appropriate skill mix, the project group should consist of researchers representing different professions, perspectives and knowledge [51]. Our project group consisted of four researchers, the authors of the article: LK, RH, AB and JD, all with health education within physiotherapy and nursing and with extensive research experience, clinical experience and higher-level teaching experience in elderly healthcare. The four authors had routine meetings throughout the process (January to September 2021) and carried out the analysis together through four workshops in order to discuss the analysis, the emerging findings and their interpretations.

### Deciding what is relevant to the initial interest—Phase 2

In Phase 2 we identified the population of studies on the topic. Phase 2 involved the project group agreeing on the focus of the meta-ethnography, identifying key articles, as well as deciding upon inclusion criteria and quality assessments [38]. Within the larger main project, Cross-Care-Old, we decided through the first workshop to focus the synthesis on the users’ perspectives regarding challenges in quality transitional care. The twelve articles, written between 2019 and 2021, represent four different contexts in the Norwegian healthcare system (see Table 2). The articles are related (they complete each other) but do not overlap, and once synthesised, we believe they will generate additional new knowledge and understandings. In addition, we found the 12 articles to be a manageable number in terms of volume, timescale and size of the project group [63]. Thus, a formal, systematic search was not required.

**Table 2:** Informants, contexts and methodologies of the 12 articles

Article	Informants	Context	Sample	Methodology	Analysis
Hestevik (2019) [39]	12 HCPs from hospital 11 HCPs home care	Two hospitals and five home care units	Purposive	Semi-structured interviews Descriptive interpretative	Thematic analysis
Hestevik (2020) [40]	15 older patients (≥ 65) 9 informal caregivers	Two hospitals and five home care units	Purposive	Semi-structured interviews Descriptive interpretative	Thematic analysis
Hestevik (2020) [41]	12 HCPs from hospital 11 HCPs home care	Two hospitals and five home care units	Purposive	Semi-structured interviews Descriptive interpretative	Thematic analysis
Kvæl (2019) [42]	15 older patients (≥ 65) 15 informal caregivers	Three IC institutions (6 wards)	Purposive	Semi-structured interviews Critical realism	Thematic analysis
Kvæl (2019) [43]	18 HCPs = three multidisciplinary team	Three IC institutions (6 wards)	Purposive	Semi-structured interviews Critical realism	Thematic analysis
Kvæl (2020) [44]	14 initial family meetings in IC services	Three IC institutions (6 wards)	Purposive	Observation of meetings Critical realism	Thematic analysis
Lilleheie (2020) [45]	18 older patients (≥ 80) 12 informal caregivers	One hospital, home care and IC	Purposive	Semi-structured interviews Phenomenological	Thematic analysis
Lilleheie (2020) [46]	12 informal caregivers of geriatric patients	One hospital, home care and IC	Purposive	Semi-structured interviews Phenomenological	Thematic analysis
Lilleheie (2020) [47]	18 older patients (≥ 80) in transitional care	One hospital, home care and IC	Purposive	Semi-structured interviews Phenomenological	Thematic analysis
Olsen (2020) [48]	20 HCPs 3 key informants and 22 meetings	A quality improvement collaborative	Purposive	Interviews and observation Social constructivism	Thematic analysis
Olsen (2021) [49]	20 HCPs 3 key informants and 22 meetings	A quality improvement collaborative	Purposive	Interviews and observation Social constructivism	Thematic analysis
Olsen (2021) [50]	20 HCPs 3 key informants and 22 meetings	A quality improvement collaborative	Purposive	Interviews and observation Social constructivism	Thematic analysis

To assess the rigour, credibility and relevance of each article, we used the critical appraisal skills programme (The CASP). This programme provides a checklist (score 1–10) and was chosen due to its systematic process for identifying strengths and weaknesses and its ease of use [64]. All the articles were considered to be of high quality (range 8–10) and conceptual relevance, thus, during analysis, we decided to order the studies alphabetically [51].

### Reading the studies—Phase 3

Phase 3 implied close reading of the identified articles to be included in the meta-ethnography [38]. First, the authors repeatedly read the articles in order to become as familiar as possible with their concepts and metaphors [51] before discussing details during the second workshop. Second, the first author extracted contextual information from the primary studies (see Table 2). Third, we extracted raw data for synthesis, that is, first- and second-order constructs [65].

Britten et al. (2002) distinguish between first-, second- and third-order constructs. First-order constructs come from the participants’ quoted input in each paper; second-order constructs represent the primary authors’ interpretations of the participants’ quoted input (themes or concepts); third-order constructs are higher-order interpretations from a tertiary analysis of the first- and second-order constructs as conducted by the project members [66]. We decided to make a one-word file with verbatim extracted raw data from all the primary studies. The authors then coded concepts independently. Furthermore, we made a list of the primary studies’ metaphors and themes, in addition to title, aim, context and conclusion.

### Determining how the studies are related—Phase 4

In Phase 4, the authors decided how the included articles may create a larger understanding than the separate parts may provide alone [38]. Regarding the aim of this meta-ethnography, the studies are related due to their similar focus on quality in transitional care from the perspectives of older patients, informal caregivers and HCPs.

Although the articles explore different contexts, they are interlinked and highly relevant locations within the clinical patient pathway. In all the primary studies, data were collected in the largest urban city of Norway. The studies have similar methodologies in terms of sampling, method of data collection and analysis. A difference appears, however, when it comes to epistemology and the use of theory during analysis. Based on the thematic analysis of themes identified in Phase 3, all authors brought ten independently extracted themes into the third workshop for discussion. According to the literature, this is a suitable approach when handling many codes and concepts [51, 52, 63].

We continually compared emerging themes with the list of the primary studies’ metaphors and themes. Furthermore, we merged the first identified themes across the primary articles into describing groups. We juxtaposed articles, and our first theme list was discussed and slowly clarified by collapsing, deleting and changing the wording. Through plenary discussion and negotiation, we identified ten categories during this phase. These included, for example, individualised care, complex geriatric problems and working conditions of staff. The text within each group formed the start for reciprocal translation in further analysis [63].

### Translating the studies into one another—Phase 5

Phase 5 involved translating the studies into one another. This required comparing the metaphors and concepts from the individual studies with each other [38]. All authors were involved in the translation. We also determined that the included articles were connected in focus in order to conduct reciprocal translation. According to Noblit and Hare (1988), reciprocal translation ‘requires the assumption that the studies can be “added” together. That is, they are clearly studies about some similar things’ ([38] p. 40).

We arranged the primary studies alphabetically. Our approach to translation was then to compare the themes and concepts developed through our thematic analysis, article by article. Beginning from our ten categories (but remaining open to new ones), we compared the themes from article 1 with article 2, the synthesis of those two were then compared with article 3, and so on. For each article, we explored issues in light of a certain theme. During this process, our initial grouping was refined as our understandings developed. Although some new sub-categories emerged, no new major categories were established during the process of translation. It is important to be aware that, even though supported in the literature, this pragmatic approach of prior grouping may have influenced the results [63].

### Synthesising translations—Phase 6

During this phase, the idea was to proceed the reciprocal translations into a line of argument synthesis [51], that is, to make the whole into something more than the parts can provide alone [38], in order to develop a higher-order analysis or third-order constructs [66]. During our final workshop, we structured the refined themes with explanations and sub-categories while juxtaposing with the primary studies’ second-order concepts or themes. This iterative process continued until the project group had developed four main themes and a line of argument synthesis. Table 3 provides an overview of the final process. The final themes represent four superior challenges within our healthcare system to achieving high-quality transitional care for older persons.

**Table 3:** Synthesising the translations

Individualised care	Person-centred versus efficient care
Predetermined patient pathway	
Relatives bridging the care gap	
Medical-oriented care	Everyday patient life versus treating the illness
Complex geriatric problems	
User choice and responsibility	User choice versus “What Matters to You”
What matters to you?	
Professional knowledge and skills	Relational versus practical care
Relational and communication skills	
Working conditions of staff	

### Expressing the synthesis—Phase 7

Phase 7 included the presentation of the synthesis in an understandable way. In our case, the audience was researchers, clinicians, politicians and other important stakeholders in healthcare. To do this, it was of great relevance that we had knowledge of the recipients’ cultures in a similar way as we have expertise in the research field to be synthesised [38]. In the following sections, we will express our line of argument, underlined by first- and second-order constructs. As recommended, we have followed the eMERGe reporting guidance [52].

## Results

The final analysis resulted in four recurring themes across the four sub-projects, expressed as challenges in order to achieve high-quality transitional care for older persons and their informal caregivers, that is, balancing person-centred versus efficient care, balancing everyday patient life versus the treatment of illness, balancing user choice versus “What Matters to You” and balancing relational versus practical care.

### Balancing person-centred versus efficient care

Balancing person-centred versus efficient care emphasises the difficulty of providing personal, tailored care in a system predominantly structured for efficient patient flow. The goal of care was, according to all informants, to contribute to a meaningful life. Although the services are supposed to be holistic and involve the older persons and informal caregivers in decisions about their care, the participants instead described a predetermined clinical pathway with a lack of personal choices regarding both time and place of services, such as hospital discharge, IC services and home care. The patients and informal caregivers called for better information, continuity and predictability, while the HCPs experienced limited professional discretion and structural barriers in daily care to meeting the patients’ needs and preferences [39–50]. Efficient patient flow issues often challenged the pathway. One HCP said,

I understand that society has to think about money, both the hospital and municipality. But there are too many cases where the patients are not heard and directly ignored because it costs NOK 5000 [550 USD] extra per night [in hospital] ([49] p. 8).

Lilleheie et al. stated that patients were commonly discharged from hospitals at an early stage, not always involved in decisions regarding the next level of care and subject to scant information reporting in a system not integrated thoroughly around a patient’s own journey. Within this system, the patients’ vulnerable conditions in the first 30 days after discharge made it the informal caregivers’ responsibility to bridge the gap [45–47].

For the informal caregivers, these expectations from the system, often outside their comfort zones and areas of expertise, and the absence of information, made the coordinating role burdensome and unpredictable. One of the informal caregivers expressed it like this:

The system is not self-explanatory and I didn’t really know anything, what to ask for and so forth. They should have given her [the patient] better information. Relatives and patients need better information ([46] p. 6).

Kvæl et al. found that IC services are highly appreciated, bridging the pathway between hospital and home; however, the method of providing these services depends highly on collaboration between the districts and each IC unit within the bureaucratic purchaser-provider model, ranging from very good to non-existent. The informants argued that in order to achieve PCC, HCPs need to be acknowledged for their work, an important outcome of PCC that in many cases was absent [42–44]. In light of the fast-in and fast-out model of hospitals and IC, the participants painted a picture of a standardised system in which the patient must fit the system, instead of a service providing help in accordance with the individualised needs and preferences of the patient, that is, PCC. One district coordinator emphasised this:

I understand the importance of the patient’s voice being heard. No one fits into a box, I understand the thinking. But I see that real patient participation [and PCC] is difficult to achieve in a health-care system that is built up like ours. These are the services we have! The day center has a specific structure that you have to fit within. And within IC there are other criteria. As a patient, you might say what you would like, but whether you ever receive it, is not up to you ([43] p. 925).

Within the context of nutritional care, Hestevik et al. emphasised that HCPs, older patients and informal caregivers expressed that individualised care was challenging to achieve. For example, although the HCPs had a strong focus on nutritional care when caring for older patients, lack of time and heavy workloads were highlighted as barriers to fulfilling their mandated responsibilities, resulting in inadequate nutritional care that consisted mainly of providing nutritional drinks and pushing the button on the microwave. Hence, nutritional assessments conducted in hospitals were seldom followed up at home [39–41].

Furthermore, the participants receiving home care reported short visits with little time for conversation about patients’ nutritional concerns or preferences, as well as frequently meeting different personnel daily. They experienced unstructured services to support the adaption of healthy food consistent with their health problems, creating gap in standard services and real PCC. One older patient described her nutritional situation in this way:

I had to get a microwave oven, because that was the first thing they [homecare services] asked for when they started coming here. I don’t like such food. I want to make homemade food. I don’t know what kinds of food I am supposed to buy. I can’t eat only ready-made meals. First of all, I can’t stand the taste of these ready meals, and secondly, I find them too expensive ([40] pp. 6-7).

In a quality improvement collaborative, HCPs from hospital care, IC and homecare illustrated that the successful crossing of knowledge boundaries was essential in practicing person-centred transitional care for older people. Knowing the patients and knowing each other, both within care teams and across levels, seems to be of great relevance for establishing a common ground in understanding patients’ personal journeys and thus promote continuity of care [48–50]. One HCP from home care emphasised the importance of this knowledge exchange:

That the user doesn’t have to repeat herself at each new place, but feels like the people I relate to here, they communicate, they know who I am [...] that the user feels like when they ask me questions they ask them as if they already know me a little ([50] p. 9).

### Balancing patients’ everyday lives versus the treatment of illness

The dominant biomedical approach in healthcare represents an insufficient understanding of the patients. Our informants asked for more holistic healthcare in order to understand how a disease influences older patients and how to reply to the accurate needs of the person, as well as the importance of considering the complexity of geriatric care for both patients and caregivers and the need to offer patients meaningful choices. In all the sub-projects, the researchers argued that HCPs need to consider aspects of everyday life with a chronic disease and expand existing resources to provide what is important to patients [43–50].

Beyond biological losses, ageing often implies noteworthy life changes, such as new roles and movement in social status. The studies examined here all focus on the shortcomings of biomedical-oriented care for older people. The HCPs in Hestevik et al. noted that the biopsychosocial dimensions of care need to be addressed to prevent, for example, depression and loneliness [39, 41]. Patients and caregivers reported fears of functional decline, with impacts on self-identity and mental health. Some people reported not liking to eat alone [40].

The importance of multidimensional healthcare is highlighted in Kvæl et al. [43]. In this study, HCPs explained that patient participation in IC services should be part of a holistic process that considers the complexity of geriatric problems, i.e., physical, social and psychological aspects. It seems like service provision is mostly based on physical criteria, at the expense of psychological and social circumstances. A nursing assistant suggested,

We see the disease but not the person behind it. I believe that if we increase our holistic understanding, it will also become more natural for us to let the patients decide ([43] p. 926).

The informal caregivers in Lilleheie et al. [46] claimed that hospital stays were framed within a medical approach, with less focus on people’s thoughts and complicated issues. One said,

It’s going to be like this: ‘Now we have arranged the pills, and the situation with the pills is OK and stabilised, so now you can go home, because we must have your bed for someone else’ ([46] p. 5).

Another patient quoted in Lilleheie et al. lamented the lack of a broader perspective: ‘They are more interested in tangible things’ [59 p. 7]. Many patients reported that an isolated life resulted in depression, a feeling of hopelessness and lack of zest for life. The narratives of HCPs illustrated complex geriatric problems. These individuals reported that giving nutritional care to many older persons was demanding due to the complexity of old-age issues. Multifaceted issues such as dental problems, cognitive impairment, depression and loneliness influence people’s appetites and food intake [39]. One HCP said,

It seems like the lack of food intake is not solely due to illness, but it is also due to more psychosocial issues like the loss of a loved one, suddenly being alone and maybe not being able to get out of the house ([39] p. 4).

The patients and family caregivers suggested that many of the staff lacked knowledge about their preferences. The staff were often students working part-time while studying in other fields than healthcare [40]. All the articles [39–50] showed that HCPs cannot fulfil their potential if the care environment is not conducive to having a suitable skill mix, cultures promoting patient involvement, healthy staff relationships and supportive organisational structures framed within a physical environment that facilitates participation. Lilleheie et al. [47] examined everyday life after hospital discharge and found that these patients described no longer having the ‘energy’ to initiate daily activities, such as house cleaning, grocery shopping and socialising. ‘Previously I had guests all the time, but I don’t have the same capacity anymore […] It has gone steeply downwards with my social life’ ([47] p. 8).

### Balancing user choice versus “What Matters to You”

Balancing user choice versus “what matters to you” embraces the tension between authentic participation and the challenges of achieving this through the structured what matters to you (WMTY) framework. Many patients experienced an absence of involvement, which could cause distress, where decisions regarding nutritional care were made over their heads. One patient remarked, ‘They have started to prepare sandwiches for me, but I have told them to stop doing that. I can manage on my own’ ([40] p. 8).

According to Kvæl et al. [42], geriatric patients and their relatives lamented a ‘lack of choice and expectation of compliance’ and ‘being perceived as deserving’. One patient had applied three times for a place in long-term care due to functional decline. However, the patient was declared not ‘sick enough’, although she had been in and out of hospitals recently.

The patients most satisfied with the quality of care were those with uncomplicated problems, adaptable personalities and socioeconomic resources. A patient’s son, aged 63, said, ‘She is probably a nice patient because she does not complain or set too high demands, and because she accepts things’ ([42] p. 7). Not all patients were interested in user choice and responsibility; some wanted to leave these aspects to the experts. However, most of them, wanted to be asked about their opinions. As a 91-year-old woman said, ‘To me participation is important, but I do not think it applies to all patients, not everyone is interested in or able to follow what is being done with them’ ([42] p. 8).

Not all patients had a choice, however. Several informal caregivers noted that discharges were made against their wishes and occurred earlier than expected. The informal caregivers missed, after discharge, the follow-up care given by HCPs in the hospital, which mainly was tailored to reflect the choices of elderly patients [46]:

We already know that patients may not be in the hospital if they are not sick and need treatment … Then we asked: ‘What about a stay or rehabilitation in another department?’. …We were a little unsure of her condition, how she was going to be when she got home. But then we really only got a message the day she was sent home, that now she had been sent home ([46] p. 7).

To explore patients’ perceptions and experiences of what matters to them regarding healthcare can support personal goal setting, in keeping with this study’s ideal that those receiving healthcare should be allowed to determine their lives, the ‘What matters to you?’ (WMTY) question was highlighted by Olsen et al. [48]. In this study, the HCPs regarded WMTY not as an ordinary question, but rather as a complicated activity requiring skills in order to be used appropriately in clinical practice. Furthermore, the participants [48] agreed that the WMTY question was complicated and occasionally troublesome to use. They described the question using words such as ‘too big’, ‘dangerous’ and ‘soaring’.

The WMTY question was in some cases understood too literally, especially when used as the initial question upon hospital admission. One participant said,

One has to try to make the question less dangerous; ‘how shall we plan ahead? What matters to you? What do you want in the future?’ So, I think it needs to be rendered less dangerous. And if your focus is on user involvement, then it really is just a part of a conversation and a larger approach ([48] p. 6).

Consequently, the informants reported the potential for building ‘castles in the air’ when using WMTY. The framing of WMTY was also based on the experience that older people often required help to respond to the question. The timing of the question was the most salient issue. Many of the informants emphasised that WMTY involved supporting the older person to identify goals that HCPs were able to help them with [48]. Kvæl et al. [42] reported too little focus on meaningful engagement with patients and caregivers during IC stays and care transitions. They suggested that the WMTY question be asked as part of the family meetings in IC to highlight the older person’s goals, preferences and resources.

### Balancing relational versus practical care

Balancing relational versus practical care embraces the dilemma HCPs may experience when juggling between individual needs, resources and organisational structures. However, as revealed in the included articles, practical tasks are often prioritised at the expense of relational care, i.e., time to engage with patients and their informal caregivers. Relational competence was described as being able to listen to other people in a reflective way, being empathetic, as well as knowing the patient. Examples of practical care were reported as working in an instrumental way and were related to technical tasks and standardised routines that HCPs provided during daily care. In addition, there must also be a greater emphasis on the work conditions of the HCPs in hospitals, IC services and home care [39–50].

To be professionally competent, the two competencies are intertwined and complementary in all clinical encounters between HCPs and patients. Hestevik et al. revealed that HCPs in home care were aware of the importance of establishing alliances with the patients and their families to recognise their will and thoughts about nutritional care. One HCP stated, ‘We work with the psychology behind it now. How can we turn food and drink into something positive?’ ([39] p. 5). They often experienced, however, insufficient time and resources to do this in daily care [39, 41]. Thus, patients and relatives believed that the HCPs lacked competence in nutritional care since they never asked for patients’ preferences regarding nutritional care or reasons for these issues, nor did they talk to them about potential solutions [40]. One patient said,

For many people living alone, the only human conversation they have during a day is with the people from home care services. I think it is important that they take the time to talk to these people, not just rush in, make food and goodbye ([40] p. 7).

Furthermore, closely related to relational care is knowing the patient, i.e., understanding their life situation and family network, daily habits, preferences and way of living [48–50]. Olsen et al. illustrated that HCPs working in specialist and primary services may see the older persons differently since hospital and IC staff only have a momentary picture of the patient, and therefore they are not always able to see a patient’s overall resources and ability to manage life after discharge [50]. One HCP in institutional care underlined this:

We write a lot of electronic reports based on our experience of how we see the patient when he is admitted here. And then maybe we see a frail older person and we haven’t looked into how they are able to function at home. And then immediately we think ‘oh they need a nursing home placement, oh poor person’ ([50] p. 8).

Additionally, in IC services, knowing the patients and their informal caregivers as well as each other, that is, establishing multiple alliances, is crucial when balancing relational and practical care. In addition, competence within rehabilitation, how to facilitate patient activity without simultaneously forcing patients as well as informal caregivers into ‘desirable behaviour’, stands out as highly important, i.e., balancing user choice and user responsibility. To obtain this balance, HCPs must develop interpersonal skills such as how to communicate at different levels using both verbal and non-verbal interactions in a sensitive way to negotiate mutual solutions [42–44]. One informal caregiver described it like this:

It’s about empowerment. In this case, you must give the patient an understanding and confidence that their reflections are necessary to hear. Being able to initiate a good dialogue ([42] p. 8).

According to Lilleheie et al., this was also the case for informal caregivers who constantly had to balance caregiving for their loved ones with both expectations from HCPs and other life commitments [45–47]. In fact, the patients in Lilleheie et al. described that in order to handle daily living, the patient was depending on informal caregivers. Statements like ‘I don’t understand how I could manage without their help’ and ‘My son organizes everything for me’ ([47] p. 8) emphasise this dependency on informal caregivers. However, the patients also had concerns that this dependency could damage their relationships [45–47].

The tendency for task orientation is addressed by Olsen [48–50]. This instrumental competency must be balanced with relational competency if care is to be person centred. Professionalism is not only the job you do—it is how you do the job. For example, Olsen et al. found that framing WMTY as a functional approach was to a large extent about supporting the other to identify physical goals that HCPs could transfer into practical tasks, often connected to user responsibility and the ability to return home with as little help as possible. Thus, it was closely linked to quality domains like efficiency. On the other hand, framing WMTY as a relational approach includes knowing the person, promoting dialogue and establishing an alliance in order to give the patient a personal voice. One participant said the following about the WMTY question:

We would very much like to hear the user’s voice and take it into account. And to get to know what matters to the user, we actually have to ask, if not, we are just guessing. And then, it is easy that we guess based on what matters to us instead ([48] p. 9).

Framing WMTY in a structural conversation, such as family meetings, might be successful. However, it is not possible to control these meetings based solely on a standardised checklist; thus, understanding our power as HCPs in relation to vulnerable patients is essential. A professional flexibility within the predefined rules of conduct may promote participation among older persons and caregivers [44]. Accordingly, balancing relational and practical care highlights the need for competent HCPs who have the ability, i.e., professional discretion, and skills to manage the multiple contextual and attitudinal factors in the practice environment and to facilitate the processes that keep the person at the centre of interactions [39–50].

### A line of argument synthesis

Quality transitional care reflects a dedication to provide the best care to patients and their informal caregivers, as well as a commitment to the team and the organisational culture. However, as our results indicate, dilemmas might occur due to conflicting policies expressing various domains of the quality concept in transitional care for older person and their informal caregivers, i.e., balancing standardisation and individualisation in transitional care pathways.

**Fig. 1:**
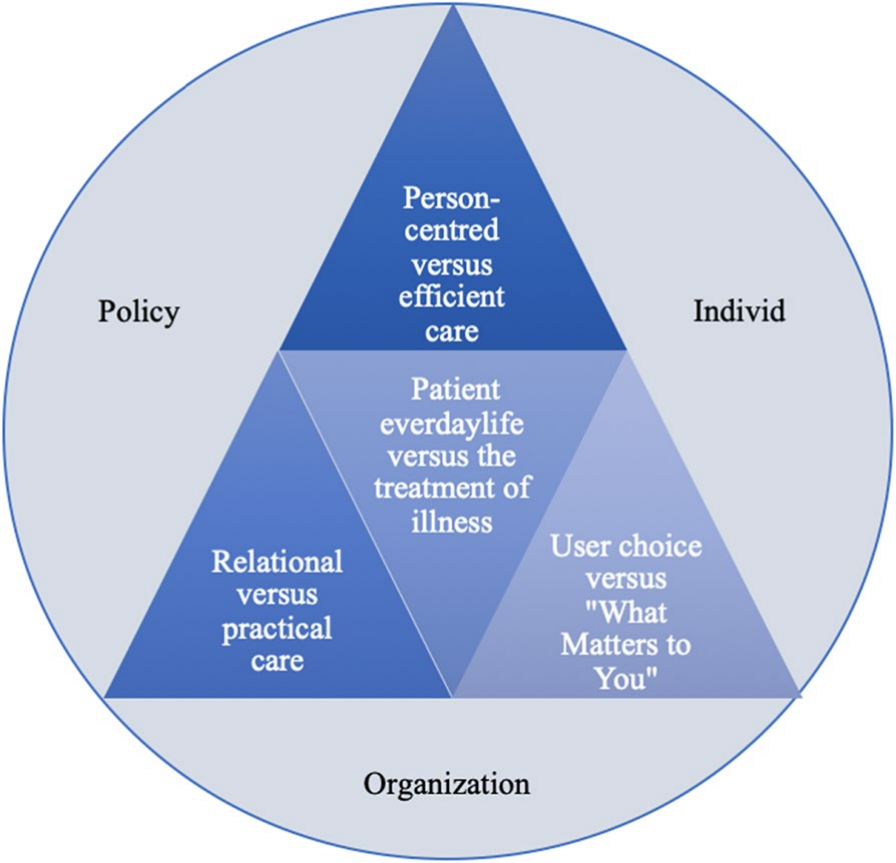
Model for conceptualisation of the experienced challenges when balancing standardisation and individualisation in transitional care pathways for geriatric patients

Figure 1 illustrates that in order to provide high-quality transitional care, the healthcare system must be balanced with respect to person-centred and efficient care, how the care team approaches everyday patient life while treating the illness, translating what matters to patients into users’ choices and relational versus practical care. Balancing these potential dilemmas must be addressed at all levels, including the policy, organisational and individual levels. The concepts within each theme are not ideal types existing in pure forms but are a method of conceptualisation used to organise theoretical and practical issues. In the same way, the various themes are integrated in transitional care. In reality, they overlap, or even compete, but when balanced, they have the potential to promote quality transitional care.

## Discussion

The aim of this article was to synthesise studies from a larger project exploring older patients, informal caregivers and HCPs’ experiences of and views on challenges to achieving high-quality transitional care. The main findings in this meta-ethnography indicate four major challenges to achieving high-quality transitional care for older persons. These challenges represent a field of tension at the policy, organisation and individual levels based on partial competing assumptions on PCC-inspired individualisation endeavours and standardisation requirements in transitional care.

In line with our results, standardisation is experienced as an essential goal of care pathways [67, 68]. Standardisation can be defined as ‘the process of agreeing upon and implementing uniform procedures, processes, designs or practices that can increase compatibility, interoperability, safety, repeatability and quality’ ([69] p. 111). Once developed and implemented, the care pathway should function as a predictable and structured multi-disciplinary care process [70]. Of relevance to this meta-ethnography is thus the challenges HCPs face when having to perform more or less standardised tasks to fulfil professional and organisational requirements for good practice. This is especially related to predefined, time-controlled tasks within daily clinical encounters, e.g., morning care, family meetings and/or help in preparing meals, reducing HCPs’ ability to adjust care to patients’ changing needs. Recent changes in practice environments related to the demand for efficiency and standardisation may negatively affect the amount of time an HCP can spend with a patient, and thus their ability to know the patient [71]. Furthermore, to secure homogenous and evidence-based care both within and across healthcare sectors and to secure fulfilment of quality improvement goals, aspects of the care process have to be made measurable [72].

It is evident that the involved actors in our synthesis have an overall and expressed agreement of the patient’s right to participate in their care. The patient’s point of view is acknowledged regarding their choices during their care episodes, discharge planning and choices that impact their life situation. However, several conflicting considerations pull the involved actors in other directions. We have uncovered that transitional care for older persons is characterised as being a balancing act in which patients, their family caregivers and the HCPs struggle to manoeuvre between conflicting considerations and discourses. Our analysis identified that standardisation relates to i) policy and organisational frameworks, ii) the limiting biomedical treatment approach of illness and iii) standardised procedures that guide practical care. Everyone struggles to practice PCC for older people [39–50]. The challenges to building relational care and enabling PCC seem to be sacrificed on the altar of standardisation.

How patients and relatives experience the transition from hospital to primary health service, but also how HCPs experience their work, is strongly characterised by the surrounding standardised frameworks for cohesion in the patient pathway [73]. This is apparent in our review study in that the interactions between patients and HCPs often take place in standardised admission meetings and discharge meetings, and it follows established routines for referral and follow-up. Although both patients and HCPs experienced standardisation as a problem, the responsibility for the delivery of quality healthcare obviously lies within the healthcare system and HCPs.

Both PCC and standardisation are increasingly promoted and adhered to in healthcare services [74]. Establishing and maintaining standards in healthcare is often considered a useful tool that contributes to the reduction of unnecessary variation in quality of care among patients. It also contributes to fair treatment, so that equal cases are treated equally, in addition to the fact that it can reduce large costs in health services [75]. At the same time, it is clear from our review that standardisation can come at the expense of PCC and that patients, relatives and HCPs felt that the individualisation of patient care intrinsic to PCC was difficult to achieve during transfer from specialists to primary healthcare services. For example, a 10-min predefined decision on a meal preparation provides little flexibility for personal follow-up. It seems that there was limited flexibility so that significant, individual variations between patients were overlooked, which led to poorer perceived quality of the care transition. Clearly, standards, as a result of established systems of patient care during transmission, often conflict with the intentions of PCC [75, 76]. In many respects, the elements in PCC and the degree of emphasis on each element are poorly defined and agreed upon [74, 75]. It is also unclear to what extent and how PCC approaches apply to a dyadic patient-HCP encounter and within organisational or societal levels of healthcare decision making [76].

Although standardised transitional care pathways are part of a general policy orientation towards austerity measures and efficiency to meet healthcare’s current and future challenges [75, 77], scholars have pointed out that PCC is often used by authorities as a rhetorical tool to soften the presumptively negative connotations towards standardisations [75]. Such opportunistic use of PCC may have great practical ramifications for HCPs while balancing a patient’s individual preferences and standardisation. The seemingly diverging or competing agendas of standardisation and individualisation may therefore create a tension and constrain PCC [76]. Pre-defined standards can provide guidelines but also challenge professional expertise when decisions to a lesser degree are based on individual judgement [78].

A central criticism of the PCC approach as devoid of considerations about the HCPs’ work environment seems closely related to the general political emphasis on agency by downplaying structure. Brannen and Nilsen (2005) point out that by ignoring organisational or societal structures, considerations of resources are taken for granted and left out when peoples’ contributions and achievements are assessed [77]. The situation of HCPs and patients in our review may therefore be considered in view of competing rationales of standardisation and individuality when HCPs fall short of achieving a form of PCC, especially amidst organisational cultures that favour efficiency above individualised care [73].

The studies in our review also highlight HCPs’ tendency towards task orientation while attempting to balance between relational and practical care. Relationships with frail, older patients seem difficult to establish and maintain in the process after transition from hospital to home or municipal care. In the standardised organisational setup, especially in a biomedically guided healthcare environment, doing PCC-inspired relational work proves to be difficult.

Task orientation is usually a behaviour of HCPs, which is closely linked to workplace conditions such as heavy workload, competing priorities and cost-effectiveness considerations [73, 79]. Every patient is different, something which must be taken more into consideration when assigning services. Otherwise, it would only be a matter of efficiency and less about quality. In a Dutch study, nurses’ work environments, both in hospitals and municipal care [79], increased administrative workloads to ensure transparency and external accountability were out of balance. The HCPs experienced that such monitoring and documentation served an external accountability goal but hardly improved patient care. This felt discrepancy between ideals of patient care and increased monitoring and registration activities contributed to the development of task-centred care by HCPs [79]. Similarly, an Australian study on the cultural factors affecting the delivery of PCC in an hospital setting showed a tension between nurses’ intentions to provide PCC and the actual task-focussed work they ended up doing. The HCPs in the study felt that a shared, collective work environment that valued efficiency over individualised patient care made it difficult to avoid task orientation [73].

Sustained imbalance between standardisation and individualisation in the process of patient pathways, may ultimately have adverse consequences on HCPs’ work satisfaction. A discrepancy in HCPs ideals of patient care and the circumventing organisational set-up may result in a consistent and demanding work environment, which is a risk factor for burnout [80, 81]. Burnout is fatigue that manifests itself as a psychosocial and physical health issue. HCPs experiencing burnout may distance themselves from both patients and colleagues and have feelings of powerlessness, emotional exhaustion and reduced work satisfaction [82]. Research shows that burnout is common among primary care nurses [83], and HCPs are particularly at risk for developing burnout [84]. One of the reasons for such burnout among HCPs may be with the lack of awareness and control of contextual factors of their work environments [80]. Hence, the HCPs might experience powerlessness to alter the factors that contribute to poor PCC. Problems arising from the seeming lack of commensurabilities of PCC and standardisation, which appears to have more to do with the organisational set-up than person failures, may diminish HCPs’ work commitments and engender high-quality patient care [82].

Notwithstanding, the review also shows that some patients are neither able to participate nor interested in participating in their care, and often they are happy to leave it in the hands of the HCPs. Consequently, the extensive orientation towards patient participation in PCC or WMTY in transitional care may run contrary to the patients’ preferences if used indifferently. There is therefore a need for a more nuanced understanding of PCC and the involvement of HCPs in relational care under institutional constraints and standardisation.

The negative connotations of standardisations may nevertheless rest on faulty assumptions. For example, Kumlin et al. found that HCPs experienced that it was challenging to ensure safe and quality transitions for patients with multiple health problems because they lacked scripts for these patients [85]. Consequently, researchers have suggested that standardised care pathways can produce good results, both for patients and HCPs, when they are tuned carefully alongside a specified type of PCC [86]. Standardised care may also have sufficient in-built flexibility to accommodate for professional discretion and critical thinking [78]. Better possibilities for discretion, trust and team work in well educated professionals within the boundaries of standardisation may improve efficient care as well as person-centred care, e.g. by reducing duplication of assessment by different health professionals asking the same questions of the patient. In-built flexibility may approve the ability for patients to interact with HCPs and to get tailored information when they need it, also outside routine meetings. Furthermore, standardised tools, procedures and routines can be viewed as integral components of PCC; they need not be opposed to each other [74, 76].

To bridge the conceptual or de facto balancing problems between individualisation and standardisation, the concept of *individualised standardisation* has been put forward. The idea is that standardisation should be considered as a guiding framework, while individualisation is sought in the dyadic interactions between patients and HCPs [87]. In addition, it is suggested that PCC approaches need to encompass organisational cultures to avoid a one-sided focus on individualisation [76]. Perhaps unsurprisingly, involving HCPs in the development of patient pathways is seen as crucial for achieving intended PCC outcomes [88]. Standardisation is therefore not exclusively negative, but there is a need for organisational set-up that allows enough flexibility for HCPs to establish relationships with patients and make meaningful connections with their PCC orientations [81, 82]. There is a paradigm shift for many providers and achieving a new practice reality may require education, altering the rewards structure and ongoing mentoring support. Accordingly, HCPs may also need education, training and mentoring in communication techniques to optimize PCC, for example through interprofessional simulation methodology.

### Methodological considerations

We have used meta-ethnography to synthesise 12 articles focussing on challenges to the provision of high-quality transitional care for older persons. We found the methodology suitable and, although time-consuming, there were few disagreements about the themes. Meta-ethnographies have the potential to promote evidence of acceptability, feasibility and appropriateness of service interventions [52]. The four authors have backgrounds as HCPs and extensive clinical and/or research experience within the field of geriatric care. Three of the authors were involved in the sub-projects of this meta-ethnography and thus knew the material in depth, while the last author read the articles for the first time and thus promoted an analytical space to the material, addressing possible researcher bias.

To obtain transparency, we have strived to present the analysis process with clarity and in detail. The findings have been discussed and challenged in the Cross-Care-Old research group to foster accuracy or validity of the synthesis. Another strength is the four different contexts, all important dimensions of the clinical transitional pathway for older persons; additionally, a wide spectrum of older patients, informal caregivers and HCPs were interviewed or observed, painting a robust picture of the presented narrative. Finally, we have followed the eMERGe reporting guidance [52]. Regarding transferability, a limitation could be that our findings are based on articles from the same larger project conducted in Norway. In addition, the departure of this study is the perspectives of patients, relatives and HCPs. Including leaders or administrative staff might have broadened the perspective on standardisation.

## Conclusion

This meta-ethnography has revealed an urgent need for an attuned conceptualisation of the experienced tension in balancing standardisation and individualisation in PCC-inspired transitional care pathways for older patients to ensure better healthcare quality for patients and more realistic working environments for HCPs. We argue that our findings, which have uncovered several negotiations between the involved actors, are helpful, providing an in-depth understanding of the tensions between different discourses. Incorporating a certain professional flexibility within the wider boundary of standardisation may give HCPs the necessary room for negotiation to meet patient values and needs while ensuring patient flow and values such as equity and evidence-based practice. The study conveys extended knowledge about the practice of transitional care from the perspectives of older patients, informal caregivers and HCPs and will be of great relevance for researchers, clinicians, politicians and other important stakeholders in healthcare to inform the development, implementation and evaluation of transitional care in the future.

## Data Availability

Data sharing is not applicable to this article as no new data was created or analyzed in this study.

## Abbreviations

CASP: Critical appraisal skills programme
HCPs: Health care professionals
IC: Intermediate care
PCC: Person-centred care
WHO: World health organisation
WMTY: What matters to you
